# Involvement of Wnt Signaling Pathways in the Metamorphosis of the Bryozoan *Bugula neritina*

**DOI:** 10.1371/journal.pone.0033323

**Published:** 2012-03-20

**Authors:** Yue Him Wong, Hao Wang, Timothy Ravasi, Pei-Yuan Qian

**Affiliations:** 1 KAUST Global Collaborative Research Program, Division of Life Sciences, Hong Kong University of Science and Technology, Clear Water Bay, Kowloon, Hong Kong SAR, China; 2 Department of Computational Bioscience Research Center, King Abdullah University of Science and Technology, Thuwal, The Kingdom of Saudi Arabia; University of Sheffield, United Kingdom

## Abstract

In this study, we analyzed the metamorphosis of the marine bryozoan *Bugula neritina*. We observed the morphogenesis of the ancestrula. We defined three distinct pre-ancestrula stages based on the anatomy of the developing polypide and the overall morphology of pre-ancestrula. We then used an annotation based enrichment analysis tool to analyze the *B. neritina* transcriptome and identified over-representation of genes related to Wnt signaling pathways, suggesting its involvement in metamorphosis. Finally, we studied the temporal-spatial gene expression studies of several Wnt pathway genes. We found that one of the *Wnt* ligand, *BnWnt10*, was expressed spatially opposite to the *Wnt* antagonist *BnsFRP* within the blastemas, which is the presumptive polypide. Down-stream components of the canonical Wnt signaling pathway were exclusively expressed in the blastemas. *Bnβcatenin* and *BnFz5/8* were exclusively expressed in the blastemas throughout the metamorphosis. Based on the genes expression patterns, we propose that *BnWnt10* and *BnsFRP* may relate to the patterning of the polypide, in which the two genes served as positional signals and contributed to the polarization of the blastemas. Another Wnt ligand, *BnWnt6*, was expressed in the apical part of the pre-ancestrula epidermis. Overall, our findings suggest that the Wnt signaling pathway may be important to the pattern formation of polypide and the development of epidermis.

## Introduction

Life began in oceans and marine invertebrates were the first to evolve [1]. Marine larval metamorphosis is therefore more ancient than terrestrial metamorphosis. Understanding marine larval metamorphosis is the key to unmask the evolutionary history of the animal kingdom [1], [2], [3], [4]. To understand how metamorphosis is evolved in different taxa and ultimately the evolution origin of animals, we need extensive understanding on the molecular mechanisms of development in different marine invertebrate taxa. Yet, comparing to what we know about the terrestrial vertebrates and invertebrates, the development of marine invertebrates is obviously understudied.

Bryozoans, also known as ectoprocts [5], belong to the protostomal lophotrochrozoa clade, which is the third major branch of bilaterian animals [6]. The molecular mechanisms of metamorphosis in Bryozoans are largely unknown. Among the phylum, anatomical changes during the initial metamorphosis in *Bugula neritina* were previously observed in great detail [7], [8], [9], [10]. *B. neritina* larvae can be obtained in large numbers and their synchronous metamorphosis can be easily triggered [11], [12], making it a good species for study. In addition, during metamorphosis, the polypide, consisting of the lophophore, digestive tract, nerve ganglia and most of the musculature, and the cystid, consisting of the epidermis and a lightly calcified chitinous housing, are built *de novo* [8], [13], [14]. These dramatic transformations make *B. neritina* a good model for the study of morphogenesis in bryozoans.

Recently, our lab generated a transcriptome dataset from various metamorphic stages of *B. neritina* [15]. Based on the results from GO annotation and KEGG mapping, we suggested that *Wnt* signaling pathways should play a major role during *the* metamorphosis of *B. neritina*. The canonical *Wnt* pathway is activated by the binding of *Wnt* ligand to the receptor *Frizzled* [16]. The *Wnt/Frizzled* binding inhibits degradation of the key protein *β-catenin* and leads to the cytoplasmic accumulation ofβ-catenin, which is translocated into the nucleus [17]. Nucleatedβ-catenin binds with *Tcf/Lef* transcription factors and activates target genes that regulate cell proliferation [18], [19], [20]. In non-canonical signaling pathways, activation of down-stream activities is independent of β-catenin and relies on different signal transduction mechanisms [21], [22]. While the non-canonical Wnt pathways were implicated in planar cell polarization [23] and convergent extension in tissue growth [24], the canonical *Wnt* pathway is broadly used by animals, ranging from vertebrates to planarians, to pattern the primary body axis. In pre-bilaterians such as sponges, hydras and cnidarians, which have an oral-aboral axis with overt radial symmetry about it, the canonical Wnt pathway controls animal-vegetal axial patterning during embryogenesis as well as oral-aboral axial patterning during metamorphosis [24], [25]. In bilaterians, the canonical Wnt signaling has been implicated in dorsal-ventral (D-V) axis patterning as well as anterior-posterior (A-P) axis specification during embryonic as well as post-embryonic development in nematodes, planarians and various vertebrate models [26], [27], [28], [29]. In nearly all examined animals, Wnts were posteriorly expressed whereas Wnt inhibitors were expressed in the anterior pole. Such a highly conserved expression pattern together with the results from gene perturbation experiments suggested that Wnts may be important universal posteriorizing factors [30], [31]. We wondered whether or not and how the *Wnt* pathway regulates axial patterning in bryozoans. Specifically, we would like to know if Wnts expressions also bias toward the posterior end in bryozoans.

In this study, we firstly studied the anatomy of pre-ancestrula at different time points by Hematoxylin Eosin (HE) staining and Toluidine blue staining. We staged the metamorphosis of *B. neritina* into different pre-ancestrula stages (the intermediate metamorphic stages). We then preformed DAVID, an annotation based enrichment analytical tool, to identify over-represented KEGG pathways in *B. neritina* transcriptome. Finally, we profiled the spatio-temporal expression patterns of two *Wnt*s, the antagonist *sFRP*, three *Frizzled*s, *β-catenin* and *GSK3β* in different pre-ancestrula stages.

## Results

### Histology of pre-ancestrula stages

**Figure 1:**
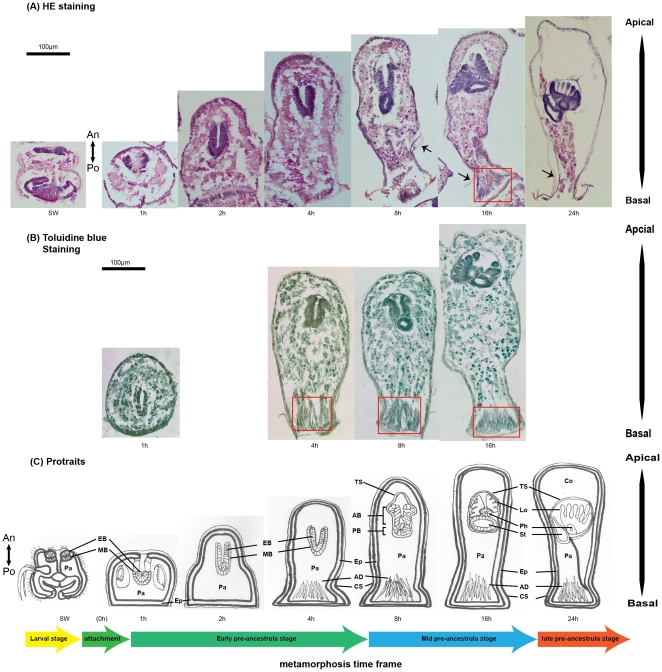
Histology of metamorphosis of *Bugula neritina*. (**A**) HE-stained and (**B**) Toluidine-stained sections of different metamorphic stages of the bryozoan *B. neritina*. The red boxes show the interlocking fibrous cells of the basal adhesion disc. Arrows point at the calcified shell. Portraits in (**C**) were modified from [7] and according to the histology of each stage. A time line for metamorphosis was defined by the overall morphology of the pre-ancestrula stage and the anatomy of the blastemas (the presumptive polypide). Note that the anterior-posterior axis and the apical-basal axis were used to represent the primary body axis of swimming larvae and pre-ancestralae respectively. AB, apical blastemas; AD, adhesion disc; An: anterior; BB, basal blastema; Ep, epidermis; Co, coelom; CS, calcified shell; Lo, (presumptive) lophophore; MB, mesodermal blastema; PC, parenchyme cells; Ph, (presumptive) pharynx; Po: posterior; St, (presumptive) stomach; TS, (presumptive) tentacle sheath.

All the time points discussed below refer to Fig. 1A and Fig. 1B. A set of portraits (Fig. 1C) modified from [8] and based on the results from histological staining shows the anatomy of *B. neritina* at various pre-ancestrula stages. The detailed histology of *B. neritina* larvae was reported in [8] and [9]. In this paper, we will refer to the primary axis of swimming larva and pre-ancestrula as anterior-posterior (A-P) axis and apical-basal axis respectively. The larval A-P axis is defined based on larval swimming direction and is corresponding to aboral-oral axis used in earlier histological studies on bryozoans larvae [7], [8], [9], [10]. In marine benthos biology, the apical-basal axis is generally used to represent the primary axis of sessile invertebrates such as hydras and sponges [32], [33]. The basal end is referred as the end where organism attached to the substrate and the apical end is referred as the end furthest from the attachment. The apical-basal axis of pre-ancestrula should not be confused with the cellular axis of epithelial cells.

Previous studies described two phases in the metamorphosis of *B. neritina*. The first phase is short, characterized by drastic morphological changes, while the second phase is characterized by the gradual development of polypide from the blastemas and the development of cystid from the internal sac. Here, we divided the second phase into three stages: the early pre-ancestrula, the mid pre-ancestrula, and the late pre-ancestrula stages.

#### The early pre-ancestrula stage (0–4 h post-attachment)

In the early pre-ancestrula stage, continuous proliferation of the apex of the epidermis results in the elongation of the body of the pre-ancestrula [9], transforming the pie-shaped pre-ancestrula at 1 h post-attachment into a tubular body at 4 h post-attachment. At 4 h post-attachment, a basal adhesion disc, consisting of fibrous longitudinal cells as shown in both the HE stain and the Toluidine blue stain, can be identified (highlighted in red boxes in Fig. 1A and 1B). A layer of shell material is also visible outside the epidermis (indicated by arrows in Fig. 1A and 1B). The blastemas are internalized during the first phase of metamorphosis, with both the epidermal and mesodermal blastemas fold inward and the epidermal blastema interior to the mesodermal blastema. The blastemas eventually become a U-shaped tissue with an apical-to-basal orientation (Fig. 1). We divided the blastemas into the apical half (apical blastemas), which develops into the lophophore, and the basal half (basal blastemas), which develops into the pharynx and the stomach. During the early pre-ancestrula stage, there is no obvious differentiation of the blastemas, except that the whole tissue increases in length.

#### The mid pre-ancestrula stage (8–16 h post-attachment)

The beginning and duration of this stage are variable. In this stage, the tubular body continues to elongate until it reached around 800 µm. The mesodermal blastema and the internalized larval pallial epithelium differentiate into the tentacle sheath, a layer of cells that separates the epidermal blastema from the parenchyme cells. The basal epidermal blastema first differentiates into a hollow sphere and subsequently into a hollow tube. Concomitantly, the apical epidermal blastema undergoes substantial proliferation and differentiation until, at 12 h post-attachment, a structure with finger-like protrusions is visible.

#### The late pre-ancestrula stage (24–36 h post-attachment)

This stage is characterized by the presence of coleomic cavity. Parenchyme cells extended from the point where the polypide attached to the basal adhesion disc. At this stage, the length of the body no longer increases. The pharynx is emerging from the region above the basal epidermal blastema below the apical epidermal blastema and the basal epidermal blastema are developing into the stomach. The apical epidermal blastema develops into a palm-like structure, with a ring of growing tentacles radiating from the base of the lophophore.

### Database for Annotation, Visualization and Integrated Discovery (DAVID) analysis on *B. neritina* transcriptome

A list of over-represented KEGG pathways is shown in Table 1. Majority of the enriched KEGG pathways are related to fatty acid or amino acid metabolisms. For instance, TCA cycle is found to be 5.99 folds over-represented and was the most enriched KEGG pathway. Several enriched KEGG pathways, such as RNA polymerase (5.07 folds enrichment), Aminoacyl-tRNA biosynthesis (3.11 folds enrichment) and Ribosome (2.83 folds enrichment) are related to translation and transcription. In term of signal transduction pathways, Wnt signaling pathways are found to be over-represented. More than two-folds enrichment is detected. 22 genes in the transcriptome are associated with *Wnt* signaling pathways.

**Table 1:** Enriched KEGG pathways identified by DAVID analysis.

KEGG Term	KEGG pathway name	Count*	P value	Fold Enrichment∧
cel00020	Citrate cycle (TCA cycle)	6	0.00235	5.99
dre00062	Fatty acid elongation in mitochondria	7	6.60E-04	5.58
mmu03020	RNA polymerase	11	2.41E-05	5.07
dre00670	One carbon pool by folate	7	0.00179	4.79
dre00630	Glyoxylate and dicarboxylate metabolism	6	0.00759	4.42
dre00280	Valine, leucine and isoleucine degradation	18	2.08E-07	4.2
dre00071	Fatty acid metabolism	14	1.15E-05	4.06
dre00260	Glycine, serine and threonine metabolism	12	1.20E-04	3.83
mmu03018	RNA degradation	18	2.64E-06	3.73
dre00640	Propanoate metabolism	12	1.68E-04	3.71
mmu03040	Spliceosome	36	1.36E-11	3.61
hsa03050	Proteasome	13	4.13E-04	3.24
dre00970	Aminoacyl-tRNA biosynthesis	12	9.54E-04	3.11
mmu00620	Pyruvate metabolism	10	0.00436	3.04
mmu00240	Pyrimidine metabolism	23	4.62E-06	2.98
hsa03010	Ribosome	21	2.84E-05	2.83
mmu00330	Arginine and proline metabolism	12	0.0027	2.82
mmu04520	Adherens junction	14	0.00677	2.29
mmu04142	Lysosome	20	0.00268	2.09
mmu04120	Ubiquitin mediated proteolysis	22	0.0025	2.01
mmu00230	Purine metabolism	25	0.00146	1.98
mmu05016	Huntington's disease	27	0.00274	1.84
mmu04310	*Wnt* signaling pathway	22	0.00743	1.84
hsa04144	Endocytosis	27	0.0065	1.72

* number of genes in the *B. neritina* transcriptome associate with the corresponding KEGG pathway.

∧ fold of enrichment (over-represented) compare to the corresponding genomic background.

### RACE and gene orthology

The full-length cDNA of *BnWnt6*, *BnWnt10*, *BnFz5/8*, *BnFz4/9/10*, *BnsFRP*, *BnGSK3β*, and *Bnβcatenin*, and the partial cDNA of BnFz1/2/7 are isolated and sequenced. The gene orthologies of *BnWnt6*, *BnWnt10*, *BnFz1/2/7*, *BnFz4/9/10*, *BnFz5/8 and BnsFRP* are supported by phylogenetic analysis using Maximum Likelihood method (Fig. S1). The full-length cDNA sequences were deposited to Genebank. Their NCBI accession numbers and blastx results are summarized in Table 2. *BnWnt6* full length transcript encoded a 339-aa protein with signal peptide (Fig S2A), 23 conserved Cys residues and two Asn-linked glycosylation sites (Fig. S3A). *BnWnt10* full length transcript encoded a 366-aa protein with signal peptide (Fig S2B), 24 conserved Cys residues, two Asn-linked glycosylation sites (Fig. S3B). *BnsFRP* was also predicted to have a signal peptide in the N-terminal. (Fig S2C).

**Table 2:** Blastx results in comparison with the full-length cDNA of corresponding genes.

Name	NCBI accession no.	Blastx results		
		Accession	Description	E value
*BnWnt6*	JN900459	ABY53107.1	Wnt6 [*Xenopus laevis*]	9E-92
*BnWnt10*	JN900460	AAC34389.1	Wnt10b [*Takifugu rubripes*]	1E-74
*BnsFRP*	JN900461	NP_571933.1	secreted frizzled-related protein 5 [*Danio rerio*]	1E-54
Bnβcat	JN900462	ADI48180.1	beta-catenin [*Crepidula fornicata*]	0
*BnGSK3β*	JN**9**00463	XP_003226831.1	PREDICTED: glycogen synthase kinase-3 beta-like isoform 1 [*Anolis carolinensis*]	4E-173
BnFz1/2/7	JN900464	NP_001124086.1	frizzled 1-like [*Danio rerio*]	4E-60
BnFz4/9/10	JN900465	NP_989429.1	frizzled-10 precursor [*Gallus gallus*]	1E-113
*BnFz5/8*	JN900466	ADZ61652.1	frizzled receptor 5/8 [*Ptychodera flava*]	3E-142

### Temporal gene expression patterns

**Figure 2:**
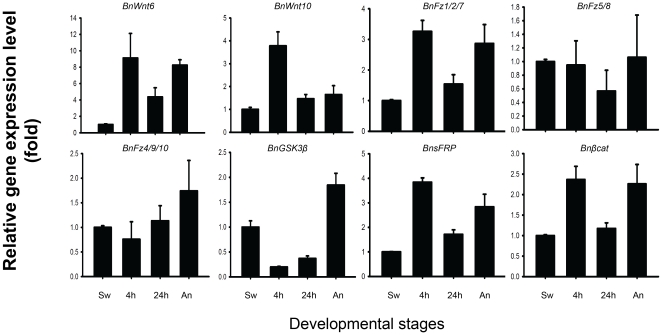
Temporal gene expression pattern of *Wnt*s, Frizzled receptors, *βcat*enin, *GSK3β*, and sFRP across metamorphosis of *B. neritina*. Gene expression levels at the swimming larval stage was taken as the baseline in every analysis.

The results of qRT-PCR assays are shown in Fig. 2. The gene expression levels in swimming larvae were taken as the base point for comparison in each analysis. Among the eight genes, the temporal gene expression patterns of *BnWnt6*, *BnWnt10*, *BnFz1/2/7*, *BnsFRP*, and *Bnβcatenin* were similar. Their expression levels peaked in the early pre-ancestrula stage, decreased in late the pre-ancestrula stage and then increased in the ancestrula stage to the same level as in the early pre-ancestrula stage. The gene expression levels of *BnFz5/8* and *BnFz4/9/10* were more stable, with less than a single-fold fluctuation, during the full metamorphosis. *BnGSK3β* was substantially down-regulated during the two pre-ancestrula stages but was up-regulated during the ancestrula stage.

### Spatial gene expression patterns

#### *BnWnt6*

**Figure 3:**
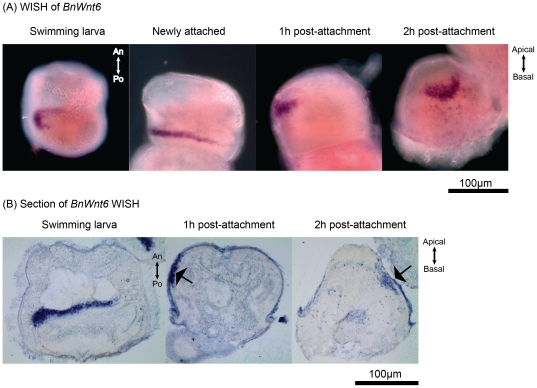
Expression *BnWnt6* in the larval and early pre-ancestrula stages. (**A**) WISH and (**B**) a representative section of WISH showing *BnWnt6* is originally expressed at the dorsal side of the wall region of the internal sac. *BnWnt6*-expressing cells become part of the epidermis on the apical half after larval attachment. An: anterior; Po: posterior.

The results of *BnWnt6* Whole mount in situ Hybridization (WISH) and a section of WISH are showed in Fig. 3. *BnWnt6* was expressed in the dorsal side of the wall region of the internal sac. During the attachment process, *BnWnt6-*expressing cells become part of the apical epidermis peripheral to the central axis. At 2 h post-attachment, *BnWnt6* expression was restricted to a small patch of cells on the apical epidermis just below the apex.

#### *BnWnt10* and downstream components of the canonical Wnt pathway

**Figure 4:**
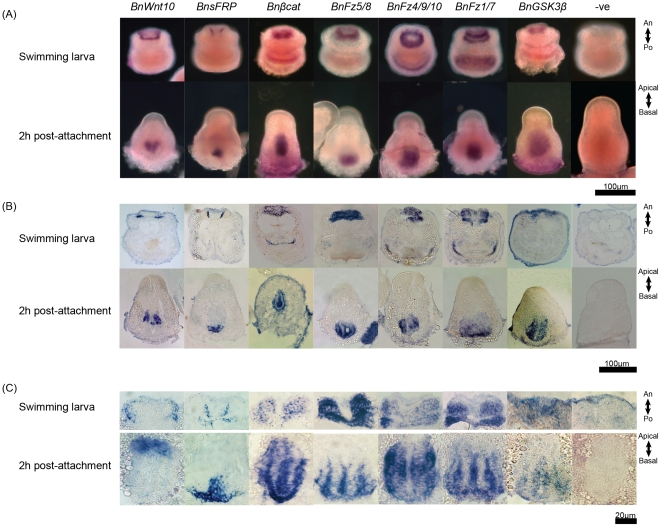
Expression of *BnWnt10*, *BnsFRP*, *Bnβcat*, *BnFz4/9/10*, *BnFz5/8*, *BnFz1/2/7*, *BnGSK3β* in the larval and early pre-ancestrula stages. (**A**) WISH and (**B**) a representative section of WISH showing *BnWnt10* is always expressed spatially opposite to *BnsFRP* while the downstream components of the *Wnt* pathway, including *Bnβcat*, *BnFz4/9/10*, *BnFz5/8*, *BnFz1/2/7*, *BnGSK3β*, are expressed throughout the blastemas. (C) Images from distinct regions with gene expression signal as shown in (B) were captured using high power (100×) objective. An: anterior; Po: posterior.

The result of WISH (Fig. 4A) and a section of WISH (Fig. 4B) for all genes examined in this study except *BnWnt6* were showed in Figure 4. In the larval stage, *BnWnt10* was expressed at the intersection between the epidermal and mesodermal blastemas peripheral to the central neural plate. *BnsFRP* was also expressed in the blastemas, but close to the central neural plate. The three Frizzled receptors, *Bnβcatenin* and *BnGSK3β*, were expressed exclusively in the blastemas but not in the central neural plate (Fig. 4C).

In the early pre-ancestrula stage (2 h post-attachment), *BnWnt10* was expressed in the apical blastemas while *BnsFRP* was expressed in the basal tip of the blastemas. Expression of *BnWnt10* decreased following an apical-to-basal gradient. The three Frizzled receptors, *Bnβcatenin* and *BnGSK3β* continued to be expressed exclusively in the blastemas.

**Figure 5:**
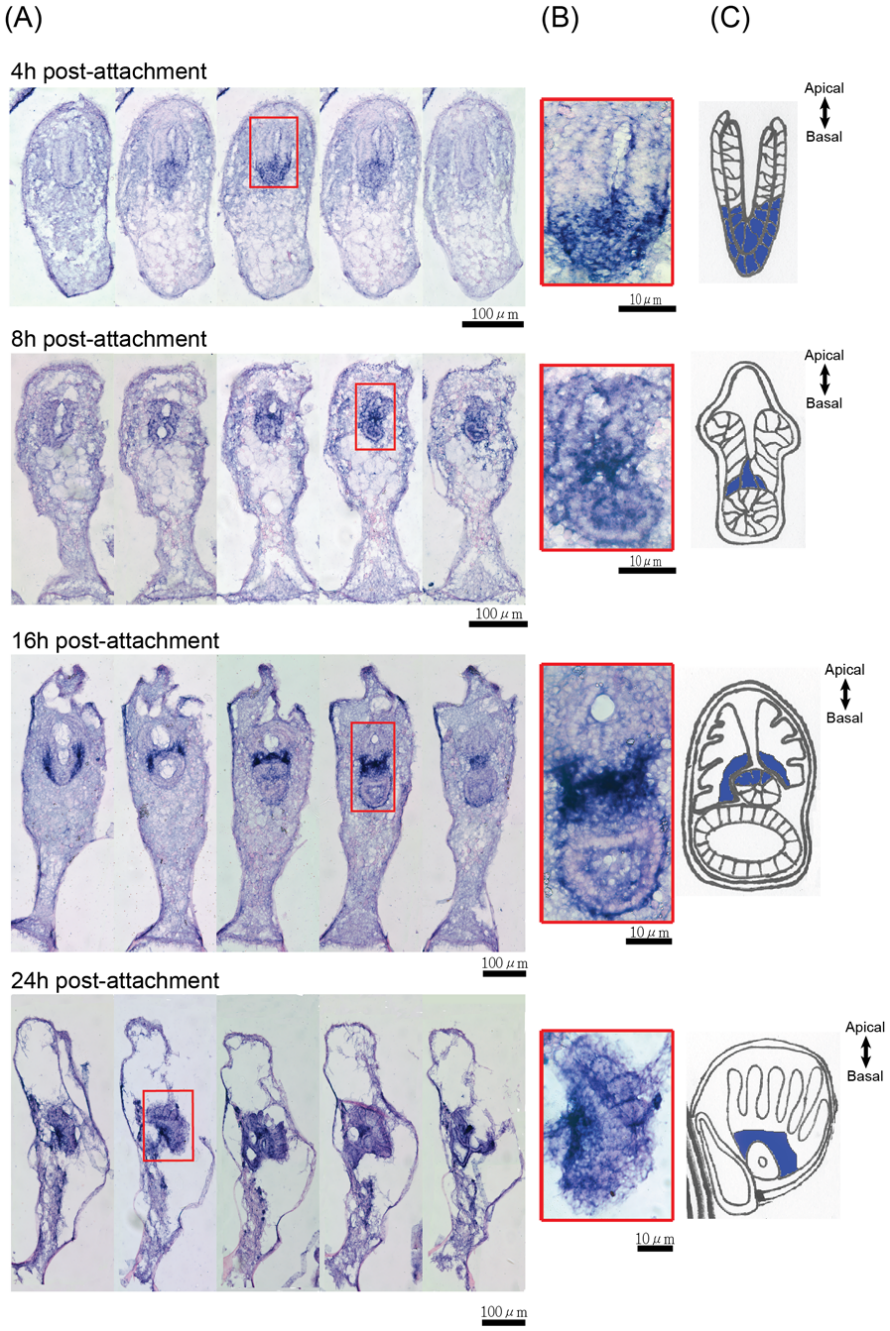
Expression pattern of *BnsFRP* in the mid- and late pre-ancestrula stages. (A) Serial sections of SISH of *BnsFRP* showing the Wnt inhibitor was expressed in distinct regions within the developing polypide. (B) Area highlighted in red box in (A) highlighted were captured using high power (100×) objective, providing the details of *BnsFRP* expression in the developing polypide. (C) Portrait depicts the anatomy of the developing polypide and the expression pattern of *BnsFRP*, which was indicated by blue color. In the early pre-ancestrula stage (4 h post-attachment) *BnsFRP* was expressed in the basal blastemas. In the mid-pre-ancestrulae stage, *BnsFRP* is expressed at the intersection between the apical and basal blastemas (8 h post-attachment) as well as in the exterior of the stomach (16 h post-attachment). In the late pre-ancestrula stage, *BnsFRP* is expressed in the base of the lophophore, but not in the pharynx or stomach.

**Figure 6:**
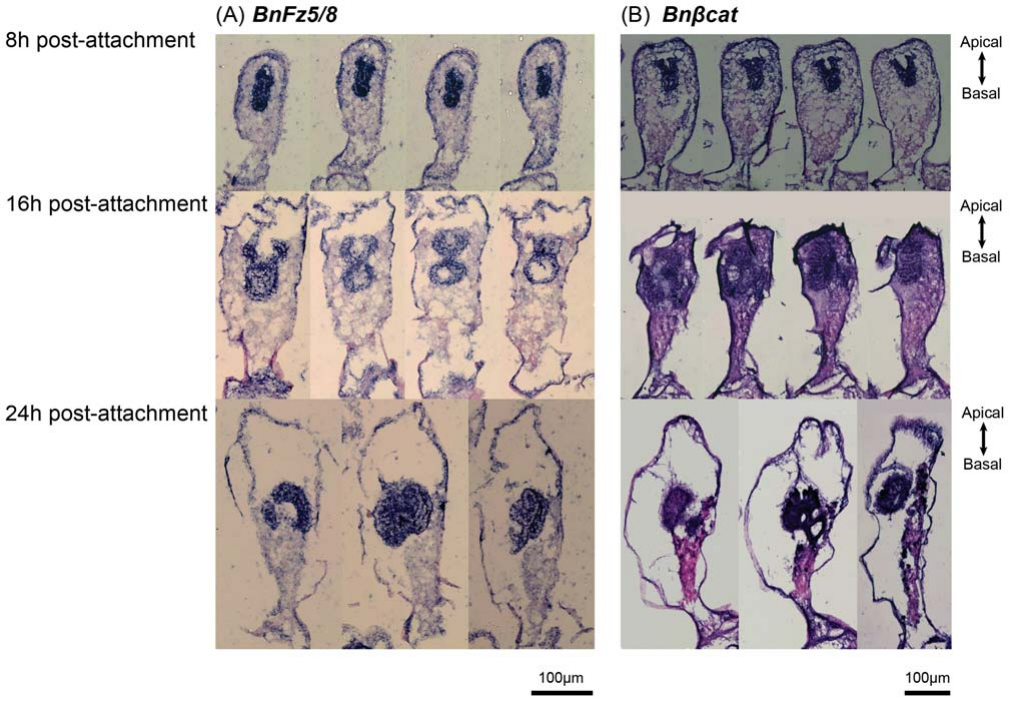
Expression pattern of (A) *BnFz5/8* and (B) *Bnβcat* in the mid- and late pre-ancestrula stages. Both *BnFz5/8* and Bn*βcat* are expressed exclusively in the blastemas during metamorphosis. Gene expressions were indicated by blue color.

**Figure 7:**
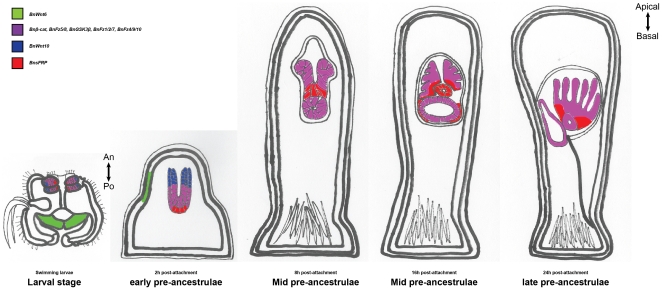
Overall summary of spatial gene expression patterns. Green: *BnWnt6*; Red: *BnsFRP*; Blue, *BnWnt10*; Purple: *BnFz4/9/10*, *BnFz1/2/7*, *BnGSK3β*, *BnFz5/8* and *Bnβcat*. Note that the purple zone in the mid- and late pre-ancestrula stages did not include *BnFz4/9/10*, *BnFz1/2/7*, *BnGSK3β*. An: anterior; Po: posterior.

Starting from 4 h post-attachment, visualization of gene expression in the interior became more difficult. We therefore semi-thin sectioned samples at 4 h (early pre-ancestrula stage), 8 h, 12 h, 16 h (mid pre-ancestrula stage) and 24 h post-attachment (late pre-ancestrula stage) before preforming in situ hybridization (refer as Section In Situ Hybridization SISH). Serial SISH sections of *BnsFRP*, *BnFz5/8 and Bnβcatenin* were shown in Fig. 5 and Fig. 6. *BnFz5/8* and *Bnβcatenin* were expressed exclusively in the blastemas in all stages (Fig. 6). *BnsFRP* was expressed at distinct region within the developing polypide at different pre-ancestrula stages (Fig. 5). At 4 h post-attachment, *BnsFRP* was still expressing at the basal epidermal blastemas. At 8 h post-attachment *BnsFRP* was expressed at the intersection between the apical and basal epidermal blastema. At 16 h post-attachment, when the digestive tract is emerging from the basal epidermal blastema in the mid and late pre-ancestrula stage, *BnsFRP* was expressed in the junction between the apical and basal blastemas and the exterior of the emerging digestive tract and but not in the basal pole of the polypide (Fig. 5). At 24 h post-attachment, *BnsFRP* expression was detectable only at the base of the developing lophophore. The overall gene expression patterns are depicted in Fig. 7.

## Discussion

In this study, we aimed at uncovering the molecular mechanisms underlying metamorphosis of *B. neritina* by analyzing genes expression patterns. A basic histological reference map is needed to correctly interpret the spatial gene expression patterns. Reed *et al.* [10] studied morphogenetic movements in the initiation phase of metamorphosis in detail. Woollacott *et al.* [7] divided the metamorphosis into the initial phase and second phase and reported the anatomy of early and advanced (late) pre-ancestrula. In this study, we divided the second phase of the metamorphosis into three pre-ancestrula stages based on the anatomy of the developing polypide and the overall morphology of pre-ancestrula. The mid-preancestrula stage, when the pharynx and stomach are developing from the basal blastemas, was not described previously. This set up a basic histological reference map for subsequent gene expression studies. In addition, we described a novel structure, the basal adhesion disc which is characterized by elongated and interlocked fibrous cells. We speculate that the adhesion disc may provide mechanical reinforcement to pre-ancestrula. Earlier histological studies on *B. neritina* did not describe this organ.

In our previous study, we sequenced the cDNA generated from various metamorphic stages of *B. neritina* using high-throughput 454 sequencing technology. A large amount of relatively low abundant but very important genes were detected and mapped to different signal transduction pathways such as Wnt signaling pathways, the Mitogen-activated protein kinase (MAPK) pathway and the cell apoptosis pathway. This led us to hypothesize that these signal transduction pathways have major roles during larval metamorphosis in *B. neritina* [15]. Yet, we did not perform any enrichment analysis to support our prediction. In this study, we analyzed the *B. neritina* transcriptome dataset using the Database for Annotation, Visualization and Integrated Discovery (DAVID) tool. The underlying principle of DAVID enrichment analysis is that, in any biological sample, if a biological process is abnormal in a given study, co-functioning genes or genes in relevant groups should have higher chance to be detected by high throughput screening technologies and hence over-represented in the corresponding gene list [34]. A gene list containing 7183 annotated contigs from the *B. neritina* transcriptome was submitted to DAVID enrichment analysis. We found significant enrichment of KEGG pathways related to energy metabolism and translation and transcription, which suggested intense energy consumption and active cellular proliferation during metamorphosis. This findings are in concert with the results from our previous proteomic analysis, which found substantial up-regulation of proteins directly involved in or indirectly related to energy metabolism and *de novo* protein synthesis [35]. More importantly, consistent with our prediction, we found that genes related to *Wnt* signaling pathways were over-represented in the *B. neritina* transcriptome, suggesting that Wnt pathways may have important functions in the metamorphosis.

The results from the qRT-PCR assay further supported the argument that *Wnt* pathways are important signal transduction pathways in the metamorphosis of *B. neritina*. The relative gene expression levels of *BnWnt6*, *BnWnt10*, and downstream component *BnFz1/2/7*, and *Bnβcatenin* substantially increased during metamorphosis while an opposite trend in the expression level of the negative regulator, *BnGSK3β*, was observed. Co-expression of *BnWnt10 and Bnβcatenin* in the blastemas suggested that the canonical *Wnt* signaling pathway is involved in the development of polypide from the blastemas. In the larval stage and the early pre-ancestrula stage (2 h post-attachment), *BnWnt10* and *BnsFRP* expressions were spatially opposite to each other in the blastemas. Throughout metamorphosis, *BnsFRP* expression was detected in the bottom part of the apical blastema, the upper part of the basal blastema and the exterior of the developing digestive tract, but never at the top of the apical blastemas.

Wnt ligands are known morphogens [36], [37] while sFRP is one of the antagonists that counteract Wnt signaling [38], [39]. The *Wnt*/*B-catenin* activity gradient as well as Wnt inhibitors such as sFRP expression act as graded positional cues to establish the primary body axis and latter direct cell specification in embryogenesis, post-embryonic development and development of multiple tissues [36], [37], [40], [41]. For instance, Wnt genes participate in epithelial-mesenchymal signaling and may specify region identity in the anterior foregut in mouse embryo [42]. Based on the spatial gene expression patterns and knowledge on the function of Wnts and sFRP in tissues patterning, we hypothesize that, as early as the larval stage, *BnWnt10* and *BnsFRP* set up a local positional signal in the blastemas. During metamorphosis, local expressions of *BnWnt10* and *BnsFRP* patterned the blastemas. *BnWnt10* expression triggers the canonical *Wnt* pathway and results in the development of lophophore from the apical blastemas. On the other hand, *BnsFRP* inhibits the canonical *Wnt* pathway activity, leading to the development of digestive tract from the basal blastemas.

It was proposed that the central role of Wnt signaling is to promote posterior rather than anterior aspects of animal tissues [30], [31]. In almost all examined bilaterians, Wnts were expressed in the vegetal pole of embryos or the posterior of larval or post-embryonic forms [30]. The A-P axis, which is the primary axis of bilaterians, defines the mouth as the anterior and the anus as the posterior [43]. Bryozoans have a U-shape digestive tract. The mouth and anus lie adjacent to each other and point at the same direction, the apical pole. Based on the development of polypide, the apical epidermal blastema, which is the precursor of the feeding apparatus lophophore, should be regarded as the anterior whereas the basal epidermal blastema, which is the precursor of the whole digestive tract, should be regarded as the posterior. The expressions of *BnWnt10* in the apical epidermal blastema and *BnsFRP* in the basal epidermal blastema are therefore right opposite to that of other bilaterians. We suggest that the role of Wnt in A-P axial patterning in the polypide of *B. neritina* may be different from that in the organogenesis in other bilaterians, with the pre-cautions that there may be additional Wnts involve in the development of polypide and we have only examined the expression pattern of one of these Wnts.

The expression pattern of *BnsFRP* in the mid and late pre-ancestrula stage suggested that *BnsFRP* may serve as an posterior signal in the emerging lophophore and, at the same time, relate to the differentiation of the pharynx, which is the anterior portion of the digestive tract. Numerous spatial expression studies have pointed out the requirement of sFRP in gut development in both vertebrates and invertebrates [44], [45], [46]. For instance, *sFRP1* and *sFRP2* might mediate mesenchymal induction of stomach epithelium in mouse [42]. Inactivation of *sFRP1* and *sFRP2* leads to reduction in fore-stomach length in mouse embryos [47]. Negative regulation of Wnt5a signaling by sFRP was suggested to control oriented cell division and apicobasal polarity in the epithelium of developing gut [47].

The exact functions of *BnWnt10* and *BnsFRP* have to be confirmed by gene perturbation experiments. If *BnWnt10* and *BnsFRP* were indeed patterning the lophophore or the polypide as a whole, perturbation of their expression should give rise to distinct phenotypes. We predict that, for example, knockdown of *BnWnt10* will produce ancestrulae with defective lophophores or ectopic digestive tracts, and vice versa for knockdown of *BnsFRP*. Unfortunately, gene manipulation techniques have not been established in any bryozoan species.

Another *Wnt* ligand, *BnWnt6*, was expressed in the apical part of the developing epidermis. The pre-anccestrula epidermis will become the living part of the ancestrula cystid and is responsible for synthesis of the chitinous calcified shell during metamorphosis. In chick embryo, *Wnt6* acts through the βcatenin–independent non-canonical pathway. And *Wnt6* expression, derived from the ectoderm, is necessary for chick neural crest induction [48]. In this study, we did not detect co-localization of *BnWnt6* and *Bnβcatenin* expression, suggesting that *BnWnt6* also acts through theβcatenin-independent non-canonical pathway. So far, there is no study reporting the function of that particular apical field of epidermal cells. Whether *BnWnt6* expression is related to the axial development of ancestrula or associated with stem cells [49] in the epidermis remains to be elucidated. Additional spatial expression analysis of *BnWnt6* on the latter pre-ancestrula and ancestrula stages may give us more hints on the possible role of *BnWnt6* in the morphogenesis of ancestrula. Interestingly, Fuch *et al.* [50] reported that another *Wnt* ligand, *BnWnt1*, was expressed in a different region of the internal sac - the neck-wall region junction. Our result could be supportive of their claim that the internal sac is regionalized and its cells might be differentially involved in adult body wall patterning.

To conclude, we have examined the gene expression patterns of various components of Wnt signaling pathways. The results suggested that the canonical Wnt pathway may be involved in the development of polypide. In particular, apical *BnWnt10* and basal *BnsFRP* expression within the blastemas suggested a possible role for the canonical Wnt pathway in patterning the polypide as a whole or only the lophophore. However, *BnWnt10* and *BnsFRP* expression patterns are different from a posterior Wnts and anterior Wnt inhibitors expression patterns observed in other bilaterians. On the other hand, *BnWnt6* was expressed in the apical epidermis. We speculate that *BnWnt6* acts through non-canonical Wnt pathway and may be associated with the development of the epidermis. This study suggested, for the first time, a possible role of Wnt signaling in pattern formation in bryozoan lophophores/polypides.

## Materials and Methods

### Larval sample preparation

Adult *B. neritina* colonies were collected from the floating rafts of a fish farm in Trio Beach, Hong Kong (22°21′19 N, 114°16′15E) between February and April, 2010. Permission to collect *B. neritina* colonies from the fish farm was given by the fish farm owner. Adult colonies were maintained in a 21°C flow-through seawater system at the Coastal Marine Laboratory (Hong Kong University of Science and Technology) for no more than 7 days before use.

Free-swimming larvae were collected according to the procedures described in our previous study [15]. *B. neritina* larvae were induced to metamorphose by placing them in the dark for one hour. Larvae that did not attach after one hour were discarded. At different time points after their initial attachment, samples of the various pre-ancestrula stages (intermediate stage of metamorphosis) specified by the number of hour(s) post-attachment and the ancestrula stage (the first zooid or juvenile, 52 h post-attachment) were collected for sectioning or whole mount *in situ* hybridization. They were first fixed with fixative and then scraped off from the petri-dish, or, for molecular studies, directly scraped off and stored under cryogenic conditions until further use.

### Specimen embedding and histological staining

Swimming larvae and pre-ancestrulae at various time points (1 h, 2 h, 4 h, 8 h, 16 h and 24 h post-attachment) were fixed in 4% paraformaldehyde in autoclaved filtered seawater for 1 h at room temperature. Samples were then dehydrated in ethanol and stored at −20°C until further use. Paraffin (McCormick Scientific, Richmond, IL, United States) embedding was performed according to the standard protocol. Semi-thin sections (7 µm) were prepared by a Leica 820II microtome (Leica instruments GmBH, Nussloch, Germany). Around 10 sections were placed on one polylysine coated slide and allowed to dry under a frame hood for 2 h. The sections were then de-waxed in xylene for 5 min and then rehydrated in descending concentrations of ethanol (100%, 95%, 90%, 75%, 50%, MilliQ water). Hematoxylin and Eosin staining and Toluidine blue staining were preformed according to [51]. Histological stained sections were then mounted on a Dpx mountant (Fluka, St. Louis, MO, United States) and visualized with a microscope under a bright-field illumination setting.

### DAVID analysis of the *Bugula neritina* transcriptome

The *B. neritina* transcriptome was blasted against the newest version of the uniprot database (released in July 2011) in the blastx mode using in-house scripts. With the E value cutoff at 1e^−8^, a total of 7234 genes had significant matches in the database. This gene list (uniport accession numbers provided in the supplementary information) was submitted to the DAVID webpage (DAVID v6.7) [52] for analysis. The detailed procedures and underlying statistics of DAVID can be found in [34] and [53]. The human (*Homo sapiens*), mouse (*Mus musculus*), zebrafish (*Danio rerio*), fruitfly (*Drosophila melanogaster*), and nematode (*Caenorhabditis elegans*) genomes were selected as the background, because these genomes have the most detailed annotations available. The results of the enrichment analysis by DAVID were filtered with the enrichment P value cutoff at 0.01 which is more stringent than recommended (≤0.05) [53].

### Rapid amplification of cDNA ends (RACE)

Total RNA was isolated from swimming larvae and samples from the early pre-ancestrula (4 h post-attachment), the late pre-ancestrula (24 h post-attachment), and the ancestrula stages using TRIzol Reagent (Invitrogen, Carlsbad, CA, United States). Total RNA extraction and cDNA synthesis procedures for the RACE template are given in [15].

Gene specific primers (GSPs) of *Wnt* pathway-related genes were designed based on the annotated sequence of the reads from the *B. neritina* transcriptome (NCBI accession number: SRA010777.2). The sequence of the reads and the corresponding gene-specific primers are listed in Table S1. The cDNA template for the 5′RACE was prepared by adding an oligo dC tail to the 3′cDNA ends by a terminal deoxynucleotidyl transferase (TdT) (USB, Cleveland, Ohio, United States) reaction. Nested PCR was performed such that the gene-specific 5′RACE1 primer and an oligodG(10)-adaptor primer were used for the first round of PCR. Gene-specific 5′RACE2 primer and adaptor primer were used for the second round of PCR. The 3′ RACE was the same as the 5′ RACE except that the cDNA template was not modified by the TdT reaction. An oligodT adaptor primer was used instead of an oligodG adaptor primer and gene-specific 3′RACE primers were used. PCR products of both 3′ and 5′ RACE were gel purified and then ligated to the pMD18-simple T vector (TaKaRa Bio Inc., Dalian, China). Transformations, insert screening, and sequencing reactions were performed as described in [15].

### Gene orthology assignment and signal peptide prediction

Gene orthology assignments for all genes were determined using the bioinformatics software Mega 5.0 [54]. Reference gene sequences from different organisms were downloaded from the NCBI protein sequence database. Prior to phylogenetic analysis, reference sequences together with the corresponding *B. neritina* genes were aligned using clusterW according to their amino acid sequences. In Wnt genes orthology assignment, mismatches in the alignment was manually corrected according to the conserved cysteine residues and two Asn-linked glycosylation sites. We used the Maximum likelihood method to conduct phylogenetic analysis. One thousand bootstraps were calculated. Signal peptide prediction was carried out by the SignalP 3.0 Server [55]

### Quantitative real-time polymerase chain reaction (qRT-PCR)

To capture the temporal expression patterns during metamorphosis, four stages – swimming larvae, early pre-ancestrulae (4 h post-attachment), late pre-ancestrulae (24 h post-attachment), and ancestrulae were chosen for qRT-PCR analysis. Total RNA extraction and cDNA synthesis procedures were the same as above except that a random hexamer primer was used in cDNA synthesis. The primer sequences are listed in Table S2. qRT-PCR assays for each gene were performed in triplicate. All of the qRT-PCR assays were carried out using iTaqSYBR Green Supermix with ROX (BioRad Life Science, Hercules, CA, United States) and were run on a Stratagene mx3000p PCR machine (Agilent Technologies, Santa Clara, CA, United States). The results were normalized using the housekeeping gene 18S of *B. neritina*. The relative changes were calculated using the 2−ΔΔCT method [56].

### *In situ* hybridization (whole-mount ISH (WISH) and sections ISH (SISH))

The DNA templates for ISH RNA probe synthesis of each gene were prepared by PCR amplification of the 3′ RACE clone using the gene-specific 3′RACE2 primer and the T7-adaptor primer. Digoxigenin-labeled probes were synthesized from the PCR-product templates according to the protocol supplied with the DIG RNA labeling kit (Roche Diagnostics, Nutley, NJ, USA).

For WISH, swimming larvae and 1 h post-attachment and 2 h post-attachment pre-ancestrulae were fixed in 3.7% formalin in AFSW overnight at 4°C. To collect the pre-ancestrulae samples, swimming larvae were allowed to attach and then fixed on a 90 mm petri dish. Briefly, the fixed samples were dehydrated in 100% methanol at −20°C until further processing. The samples were rehydrated by descending concentrations of methanol in PBS-0.1%TritonX100 (PBST). The larvae were washed and penetrated by proteinase K (New England Biolabs, Ipswich, MA, USA) treatment. Swimming larvae were incubated for 7 min while samples from the two pre-ancestrula time points were incubated for 15 min. Samples were post-fixed by 4% PFA and washed before pre-hybridization at 56°C for 1 h in a hybridization mix. RNA probe hybridization was conducted at 56°C overnight. In each hybridization, no more than a 30 ng antisense RNA probe was used. The hybridized samples were washed with hybridization mix (without sperm DNA or heparin). The samples were blocked for 1 h and then incubated in 1∶5000 antiDIG-AP antibody (Roche Diagnostics, Mannheim, Germany) at 4°C overnight with orbital rotation. They were washed and then treated with alkaline Tris buffer (100 mM Tris-HCl pH 9.5, 100 mM NaCl, 50 mM MgCl2, 0.1% Tween20) before further incubation in AP stain development solution (Roche Diagnostics, Mannheim, Germany) at room temperature in the dark. Staining was stopped by TE buffer when the samples developed a blue or purple color band. The samples were visualized under a microscope (Olympus, Tokyo, Japan) with a dark-field illumination setting. *B. neritina* samples were not fully transparent even after the WISH protocol, sectioning was necessary to visualize the precise expression pattern, especially the genes expressed in the interior of the specimen. WISH specimens were embedded in paraffin, semi-thin sectioned, de-waxed, mounted, and visualized as described above.

For SISH, pre-ancestrulae at 4 h, 8 h, 16 h, 24 h post-attachment were fixed in 4% PFA for 1 hour. Embedding and paraffin sectioning by microtome were completed as described above. Sections were de-waxed in freshly prepared xylene for 5 min and rehydrated in a descending concentration of ethanol (100%, 90%, 75%, DEPC treated MilliQ water, freshly prepared). Penetration by Proteinase K, post-fixation, pre-hybridization, anti-sense probe hybridization, probe washing, non-specific antigen blocking, antibody incubation, and stain development were the same as for WISH except all the procedures were carried out on glass slides.

## Supporting Information

Figure S1 **Maximum likelihood phylogenetic orthology assignment of (A)** ***Wnt*** **s, (B) Frizzled receptors and (C) secreted Frizzled Related Protein. (1000 bootstrap replicates).**
(DOCX)

Figure S2 **Possession of N-terminal signal peptide as predicted by SignalP 3.0. (A)** ***BnWnt6*** **, (B)** ***BnWnt10*** **and (C)** ***BnsFRP*** **.**
(DOCX)

Figure S3 **Alignment of (A)** ***BnWnt6*** **and (B)** ***BnWnt10*** **with reference sequences.** Conserved cysteine residues were bolded and highlighted in red color. Signal peptides were highlighted in purple. Asn-link glycoxylation sites were highlighted in green.
(DOCX)

Table S1 **Sequence reads from the** ***Bugula neritina*** **transcriptome (NCBI accession number: SRA010777.2) and the corresponding gene-specific primers.**
(XLSX)

Table S2 **Primers for the qRT-PCR assay.**
(XLS)

## References

[1] Ryan F (2010).

[2] Bishop CD, Erezyilmaz DF, Flatt T, Georgiou CD, Hadfield MG (2006). What is metamorphosis?. Int Comp Biol.

[3] Wilbur HW (1980). Complex life cycles.. Annu Rev Ecol Sys.

[4] Marshall DJ, Morgan SG (2011). Ecological and evolutionary consequences of linked life-history stages in the sea.. Curr Biol.

[5] Nielsen C (1971). Entoproct life-cycles and the entoproct/ectoproct relationship.. Ophelia.

[6] Tessmar-Raible K, Arendt D (2003). Emerging systems: between vertebrates and arthropods, the Lophotrochozoa.. Curr Opin Genet Dev.

[7] Woollacott RM, Zimmer RL (1971). Attachment and Metamorphosis of the Cheilo-ctenostome Bryozoan *Bugula neritina* (Linne).. J MORPH.

[8] Woollacott RM, Chia FU, Rice ME (1977). Metamorphosis of cellulariodid bryozoans.. Settlement and metamorphosis of marine invertebrate larvae.

[9] Mukai H, Terakado K, Reed CG, Harrison FW, Woollacott RM (1989). Microscopic Anatomy of Invertebrates Lophophorates, Entroprocta, and Cyciophora.. Chapter 3:Bryozoa.

[10] Reed CG, Woollacott RM (1982). Mechanisms of rapid morphogenetic movements in the metamorphosis of the bryozoan Bugula neritina (Cheilostomata, Cellulariodea). I. Attachment to the substratum.. J Morpho.

[11] Marshall DJ, Keough MJ (2003). Variation in the dispersal potential of non-feeding invertebrate larvae: the desperate larva hypothesis and larval size.. Mar Ecol Prog Ser.

[12] Lynch WF (1947). The behavior and metamorphosis of the larva of *Bugula neritina* (linnaeus): experimental modification of the length of the free-swimming period and the responses of the larvae to light and gravity.. Biol Bull.

[13] Zimmer RL, Woollacott RM (1977). Structure and classification of gymnolaemate larvae..

[14] Zimmer RL, Woollacott RM (1977). Metamorphosis, ancestrulae, and coloniality in bryozoan life cycles..

[15] Wang H, Zhang H, Wong YH, Voolstra C, Ravasi T (2010). Rapid transcriptome and proteome profiling of a nonmodel marine invertebrate, *Bugula neritina*.. Proteomics.

[16] Bhanot P, Brink M, Samos CH, Hsieh JC, Wang Y (1996). A new member of the frizzled family from Drosophila functions as a Wingless receptor.. Nature.

[17] Aberle H, Bauer A, Stappert J, Kispert A, Kemler R (1997). Beta-catenin is a target for the ubiquitin-proteasome pathway.. EMBO J.

[18] Behrens J, von Kries JP, Kuhl M, Bruhn L, Wedlich D (1996). Functional interaction of beta-catenin with the transcription factor LEF-1.. Nature.

[19] van de Wetering M, Cavallo R, Dooijes D, van Beest M, van Es J (1997). Armadillo coactivates transcription driven by the product of the Drosophila segment polarity gene dTCF.. Cell.

[20] Molenaar M, van de Wetering M, Oosterwegel M, Peterson-Maduro J, Godsave S (1996). Xtcf-3 Transcription factor mediates beta-catenin-induced axis formation in xenopus embryos.. Cell.

[21] Kohn AD, Moon RT (2005). Wnt and calcium signaling: β-Catenin-independent pathways.. Cell Calcium.

[22] Kikuchi A, Yamamoto H (2008). Tumor formation due to abnormalities in the β-catenin-independent pathway of Wnt signaling.. Cancer Sci.

[23] Katoh M (2005). WNT/PCP signaling pathway and human cancer (Review).. Oncol Rep.

[24] Yamanaka H, Moriguchi T, Masuyama N, Kusakabe M, Hanafusa H (2002). JNK functions in the non-canonical Wnt pathway to regulate convergent extension movements in vertebrates.. EMBO Rep.

[25] Adamska M, Degnan SM, Green KM, Adamski M, Craigie A (2007). Wnt and TGF-β expression in the Sponge *Amphimedon queenslandica* and the Origin of Metazoan Embryonic Patterning.. PLoS ONE.

[26] Herman MA, Horvitz HR (1994). The Caenorhabditis elegans genelin-44 controls the polarity of asymmetric cell divisions.. Development.

[27] Petersen CP, Reddien PW (2008). Smed-βcatenin-1 is required for anteroposterior blastema polarity in planarian regeneration.. Science.

[28] Christian JL, Moon RT (1993). Interactions between Xwnt-8 and Spemann organizer signaling pathways generate dorsoventral pattern in the embryonic mesoderm of *Xenopus*.. Gene Deve.

[29] Glinka A, Wu W, Delius H, Monaghan AP, Blumenstock C (1998). Dickkopf-1 is a member of a new family of secreted proteins and functions in head induction.. Nature.

[30] Peterson CP, Reddien PW (2009). Wnt signaling and the polarity of the primary body axis.. Cell.

[31] Kiecher C, Niehrs C (2001). A mophogen gradient of Wnt/β-catenin signaling regulates anteroposterior neural patterning in *Xenopus*.. Development.

[32] Wiens M, Belikov SL, Kaluzhnaya OV, Krasko A, Schröder HC (2006). Molecular control of serial module formation along the apical–basal axis in the sponge Lubomirskia baicalensis: silicateins, mannose-binding lectin and mago nashi.. Deve Gene Evol.

[33] Grens A, Gee L, Fisher DA, Bode HR (1996). CnNK-2,an NK-2 homeobox gene, has a role in patterning the basal end of the axis in hydra.. Deve Biol.

[34] Huang DW, Sherman BT, Lempicki RA (2009). Systematic and integrative analysis of large gene lists using DAVID bioinformatics resources.. Nat Protocol.

[35] Zhang H, Wong YH, Wang H, Chen Z, Arellano SM (2010). Quantitative proteomics identify molecular targets that are crucial in larval settlement and metamorphosis of *Bugula neritina*.. J Proteome Res.

[36] Charron F, Tessier-Lavigne M (2005). Novel brain wiring functions for classical morphogens: a role as graded positional cues in axon guidance.. Development.

[37] Zecca M, Basler K, Struhl G (1996). Direct and long-range action of a wingless morphogen gradient.. Cell.

[38] Dennis S, Aikawa M, Szeto W, d'Amore PA, Papkoff J (1999). A secreted frizzled related protein, FrzA, selectively associates with Wnt-1 protein and regulates wnt-1 signaling.. J Cell Sci.

[39] Bafico A, Gazit A, Pramila T, Finch PW, YanivA (1999). Interaction of frizzled related protein (FRP) with Wnt ligands and the Frizzled receptor suggests alternative mechanisms for FRP inhibition of Wnt signaling.. J Biol Chem.

[40] Cadigan KM, Fish MP, Rulifson EJ, Nusse R (1998). Wingless repression of Drosophila frizzled 2 expression shapes the Wingless morphogen gradient in the wing.. Cell.

[41] Strigini M, Cohen SM (2000). Wingless gradient formation in the Drosophila wing.. Curr Biol.

[42] Heller SR, Dichmann DS, Jensen J, Miller C, Wong G (2002). Expression patterns of Wnts, Frizzleds, sFRPs, and misexpression in transgenic mice suggesting a role for Wnts in pancreas and foregut pattern formation.. Deve Dynamics.

[43] Hickman CP, Roberts LS, Larson A (2003). Animal Diversity, 3rd ed..

[44] Nojima M, Suzuki H, Toyota M, Watanabe Y, Maruyama R (2007). Frequent epigenetic inactivation of SFRP genes and constitutive activation of *Wnt* signaling in gastric cancer.. Oncogene.

[45] Lickert H, Kispert A, Kutsch S, Kemler R (2001). Expression patterns of *Wnt* genes in mouse gut development.. Mech Develop.

[46] Leimeistera C, Bacha A, Gessler M (1998). Developmental expression patterns of mouse sFRP genes encoding members of the secreted frizzled related protein family.. Mech Develop.

[47] AMatsuyama M, Aizawa S, Shimono A (2009). Sfrp Controls Apicobasal Polarity and Oriented Cell Division in Developing Gut Epithelium.. PLoS Genet.

[48] Schmidt C, McGonnell IM, Allen S, Otto A, Patel K (2007). Wnt6 controls amniote neural crest induction through the non-canonical signaling pathway3.. Deve Dynamic.

[49] Sokol SY (2011). Maintining embryonic stem cell pluripotency with Wnt signaling.. Development.

[50] Fuchs J, Martindale MQ, Hejnol A (2011). Gene expression in bryozoan larvae suggest a fundamental importance of pre-patterned blastemic cells in the bryozoan life-cycle.. Evo Devo.

[51] Lillie RD, Pizzolato P, Donaldson PT (1976). Nuclear stains with soluble metachrome mordant lake dyes. The effect of chemical endgroup blocking reactions and the artificial introduction of acid groups into tissues.. Histochemistry.

[52] DAVID Bioinformatics Resources 6.7.. http://david.abcc.ncifcrf.gov/tools.jsp.

[53] Huang DW, Sherman BT, Lempicki RA (2009). Bioinformatics enrichment tools: paths toward the comprehensive functional analysis of large gene lists.. Nucleic Acids Res.

[54] Tamura K, Peterson D, Peterson N, Stecher G, Nei M (2011). MEGA5: Molecular Evolutionary Genetics Analysis Using Maximum Likelihood, Evolutionary Distance, and Maximum Parsimony Methods.. Mol Biol Evol.

[55] Petersen TN, Brunak S, Heijne GV, Nielsen H (2011). SignalP 4.0: discriminating signal peptides from transmembrane regions.. Nature Methods.

[56] Livak KJ, Schmittgen TD (2001). Analysis of relative gene expression data using real-time quantitative PCR and the 2(−Delta Delta C(T)) Method.. Methods.

