# Supplementary material for: Involvement of Wnt Signaling Pathways in the Metamorphosis of the Bryozoan Bugula neritina
Source: PLoS One. 2012 Mar 20;7(3):e33323. doi: 10.1371/journal.pone.0033323 (PMC3308966; doi:10.1371/journal.pone.0033323)
Supplement: Figure S1 — Maximum likelihood phylogenetic orthology assignment of (A) Wnt s, (B) Frizzled receptors and (C) secreted Frizzled Related Protein. (1000 bootstrap replicates). (DOCX) [file pone.0033323.s001.docx]

**Figure S1.** Maximum likelihood phylogenetic orthology assignment of (A) Wnts, (B) Frizzled receptors and (C) secreted Frizzled Related Protein. (1000 bootstrap replicates)

A

B

C
