# Supplementary figures and images for: Involvement of Wnt Signaling Pathways in the Metamorphosis of the Bryozoan Bugula neritina
Source: PLoS One. 2012 Mar 20;7(3):e33323. doi: 10.1371/journal.pone.0033323 (PMC3308966; doi:10.1371/journal.pone.0033323)

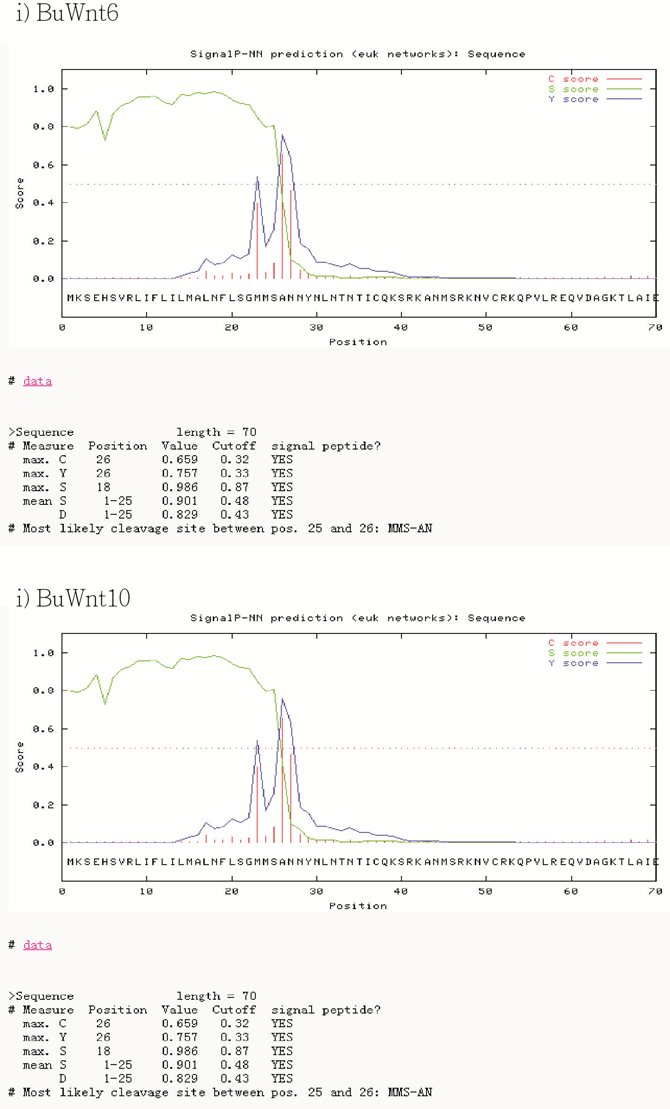

Supplement: Figure S2 — Possession of N-terminal signal peptide as predicted by SignalP 3.0. (A) BnWnt6 , (B) BnWnt10 and (C) BnsFRP . (DOCX) [file pone.0033323.s002.docx]
