# Supplementary material for: Involvement of Wnt Signaling Pathways in the Metamorphosis of the Bryozoan Bugula neritina
Source: PLoS One. 2012 Mar 20;7(3):e33323. doi: 10.1371/journal.pone.0033323 (PMC3308966; doi:10.1371/journal.pone.0033323)
Supplement: Figure S3 — Alignment of (A) BnWnt6 and (B) BnWnt10 with reference sequences. Conserved cysteine residues were bolded and highlighted in red color. Signal peptides were highlighted in purple. Asn-link glycoxylation sites were highlighted in green. (DOCX) [file pone.0033323.s003.docx]

1. BnWnt6 alignment

DrWnt6 -MGCITCKACYLPSHISYQTAMEGDWQERWAVGSPLVMDPNSI**C**RKTKRLAGKQAEL**C**QT 59

HsWnt6 MLPPLPSRLGLLLLLLLCPAHVGGLW---WAVGSPLVMDPTSI**C**RKARRLAGRQAEL**C**QA 57

DpWnt6 -MMMEPMRLCRQ-----------------RRVRQHLERTNFNDNGRHERIKSRQVEI**C**RK 42

TcWnt6 -MHAVLVSVYLLLVTPITLT--------WWAAGSQLVMDPRTI**C**KKTKRLRGKMAEI**C**SK 51

BnWnt6 MKSEHSVRLIFLILMALNFLSG-------MMSANNYNLNTNTI**C**QKSRKANMSRKNV**C**RK 53

DrWnt6 QPEIVNEVAKGAKLGVRE**C**QYQFRFRRWN**C**TSQNKYFGKILQQDIRETAFVYAITAAGVT 119

HsWnt6 EPEVVAELARGARLGVRE**C**QFQFRFRRWN**C**SSHSKAFGRILQQDIRETAFVFAITAAGAS 117

DpWnt6 EPKVLQEIVKGAQLGTKE**C**QHQFRNRRWN**C**TTARKSLRKVIARDTRETAFVNAVVAAGVT 102

TcWnt6 Q-ALVDQIRLGVELGQRE**C**QYQFRFRRWN**C**TSSRKSIRKVLLRDTRETGFVNAVLAAGVT 110

BnWnt6 QPVLREQVDAGKTLAIEV**C**QENFIDRRWN**C**TTSHSSHRKIMKTDTREAAYLNAITSAGIL 113

DrWnt6 HAVTQA**C**SMGELLQ**C**G**C**EATRSRGPP-PR--------LASMGPTEG-VKWEWGG**C**G-DDV 168

HsWnt6 HAVTQA**C**SMGELLQ**C**G**C**QAPRGRAPPRPSGLPGTPGPPGPAGSPEGSAAWEWGG**C**G-DDV 176

DpWnt6 YTVTQA**C**SSGHLLQ**C**T**C**DKTMKGVSP------------------DG--DWEWGG**C**A-DNV 141

TcWnt6 YQVTRA**C**TTGELLG**C**S**C**DRKMKSKKNKKR--------LKMASMPEG--DWEWEA**C**GGENI 160

BnWnt6 YSVTRA**C**SMGILWQXH**C**DGTKRDVASN--------------------EMWSWXG**C**X-DDI 152

DrWnt6 EFGYEKSKQFMDARRRKGKSDIRTLIDLHNNEAGRLAVKNYMRTE**C**K**C**HGLSGS**C**TLRT**C** 228

HsWnt6 DFGDEKSRLFMDARHKRGRGDIRALVQLHNNEAGRLAVRSHTRTE**C**K**C**HGLSGS**C**ALRT**C** 236

DpWnt6 QHGYKKSREFMDAKYRK-RSDLKTQVMLHNNEAGRLAVKNFMRTE**C**K**C**HGLSGS**C**TLRT**C** 200

TcWnt6 DFGLKKSKDFLDTRYKK-RSDMKTLVKLHNYVAGRMAIKNHMRTE**C**K**C**HGLSGS**C**TLKT**C** 219

BnWnt6 VYGYNKSKQFTKNKHIS--SDIKELVRNHNNEAGRMTITKNMRRN**C**K**C**HGLSGS**C**TVKT**C** 210

DrWnt6 WKKMPHFREVGDRLLERFNGASKVMGGNDGKTLIPVGQNIKPPDKQDLIYSAESPDF**C**LP 288

HsWnt6 WQKLPPFREVGARLLERFHGASRVMGTNDGKALLPAVRTLKPPGRADLLYAADSPDF**C**AP 296

DpWnt6 WRKLPLFRDVATRLKEKFDGAAKVIPGNDGKTIIPEVASIKPPGREDLIYSEESPDF**C**NP 260

TcWnt6 WRKMPPFREVGNRLKERFDGAVKVIAGNDGQSFMPEDSSIKPPGKTGLVYSEESPHF**C**LP 279

BnWnt6 WRNMPPFDMVGKALKDRYDGAPKVTGDNDGKTLIPEGKTVKPPSNLDLVYTDESPDF**C**VP 270

DrWnt6 NRKTGSLGTRGRT**C**NSTALDVSG**C**DLL**CC**ERGHRDETVVLEEN**C**L**C**RFHW**CC**VVQ**C**KK**C**L 348

HsWnt6 NRRTGSPGTRGRA**C**NSSAPDLSG**C**DLL**CC**GRGHRQESVQLEEN**C**L**C**RFHW**CC**VVQ**C**HR**C**R 356

DpWnt6 DRVTGSLGTAGRV**C**NSTSPGVEG**C**ELL**CC**GRGYETRTTKTRVN**C**H**C**RFKW**CC**EVT**C**KI**C**T 320

TcWnt6 NNTLGSFGTQGRT**C**VETSPGEEG**C**SIL**CC**GRGSRSHDETEEKN**C**K**C**KFLW**CC**EVK**C**EK**C**N 339

BnWnt6 NKKYGTSGTMGRL**C**NATSFDPDG**C**DIM**CC**NRGYERQSFQVRQK**C**R**C**KFVW**CC**EVV**C**DT**C**I 330

DrWnt6 VRKELSL**C**Q 357

HsWnt6 VRKELSL**C**L 365

DpWnt6 VKKHVNT**C**R 329

TcWnt6 ETRTIST**C**L 348

BnWnt6 NNVTIHL**C**K 339

(B)BnWnt10 alignment

HsWnt10b ---------MLEEPRPRPPPSGLAGLLFLALCSRALS----NEILGLKLPGE-PPLTANT 46

DrWnt10b ---------MELPHRQCLGRVLIVTAALLSPAFTVLG----NDILGLKVAGE-PVLTPNA 46

HsWnt10a MGSAHPRPWLRLRPQPQPRPALWVLLFFLLLLAAAMPRSAPNDILDLRLPPE-PVLNANT 59

NvWnt10 ---------MDFRG------FVKVLLIFFQIHSKGFG----YGILKTSIPFEDPVINSNT 41

PdWnt10 ---------MTVMTS---WILLWLCLAALMTHRCNGG----NDVLGLDIPTE-PNLDPNT 43

DpWnt10 ---------MRRSDLLLSSNSLMNIRRKPDEKRRQKEDKYFFNSVMERQRRK--PRPTSA 49

DmWnt10 ------------------------MAQQFDEK--DLRQTWLYGLPDGR-----------A 23

BnWnt10 ---------MEIGFLFRYQRFFIVISFLHVTYLYSLPK---NEILQFQAPRPEPTINPDN 48

HsWnt10b V**C**LTLSGLSKRQLGL**C**LRNPDVTASALQGLHIAVHE**C**QHQLRDQRWN**C**SALEGGGRLPHH 106

DrWnt10b V**C**LRLAGLTKKQMRL**C**VRSPDVTASALQGIQVAIHE**C**QHQLRDQRWN**C**SSLENHGKLPHQ 106

HsWnt10a V**C**LTLPGLSRRQMEV**C**VRHPDVAASAIQGIQIAIHE**C**QHQFRDQRWN**C**SSLETRNKIPYE 119

NvWnt10 V**C**KNTPSLSKEQLKM**C**RRLPDVVASALQGMQYAIHE**C**LAQFRYRRWN**C**SSLEMKNRNPLA 101

PdWnt10 V**C**KTYPDLTAKQYSL**C**SRYPDVTASAIQGIQVAIHE**C**QRQFKTHRWN**C**SALERKNKNPHS 103

DpWnt10 I**C**KNFPGLSKKQLEL**C**FRYPDVMSAAIGGLQLAVNE**C**QFQFQKHRWN**C**SALDRKNRNPHS 109

DmWnt10 T**C**RSVPGLTKDQVEL**C**YKASDVTAAALEGLDMAIRE**C**QIQFQWHRWN**C**SSLSTKSRNPHA 83

BnWnt10 I**C**RDFENLQPHQKAI**C**RKHPHATASGMQGILLAIHE**C**QRQFSGQRWN**C**SNLDTS--IAND 106

HsWnt10b SAILKRGFRESAFSFSMLAAGVMHAVATA**C**SLGKLVS**C**G**C**GWKGSGEQDRLRAKL--LQL 164

DrWnt10b SAILNRGFRESAFSLSLLAAGVVHSVASA**C**SLGKLRG**C**G**C**EAKRRLDDDKIRLKLTQLQL 166

HsWnt10a SPIFSRGFRESAFAYAIAAAGVVHAVSNA**C**ALGKLKA**C**G**C**DASRRGDEEAFRRKLHRLQL 179

NvWnt10 NPLLSRGFRETAFVHAILSAGMTSSVARA**C**SMGKLAK**C**G**C**DESLRGRG------------ 149

PdWnt10 SPFLARGYKETAFAYAILAAGVVTQVARA**C**SLGKLES**C**G**C**QPVLNHAT------------ 151

DpWnt10 SNFLQKGYRETAFAYAVSSAGVAHSVSKA**C**GQGKLES**C**G**C**DPKSQRG------------- 156

DmWnt10 SSLLKKGYRESAFAFAISAAGVAHSVARA**C**SQGRLMS**C**G**C**DPTINRKTLNKNLRQSLDKE 143

BnWnt10 HPLMSKGYRETAFTHAITAAGILHQVATS**C**ALGKISS**C**S**C**VNSYNDYNS----------- 155

HsWnt10b QALSR--------GK----------SFPHSLPSPGPGSSPSPGP------QDTWEWGG**C**N 200

DrWnt10b QTFQRSGVSLAGAGENTPELSSLHGSLPANLHSSHPMSLLKPLPDEVTMLQDTWEWGG**C**S 226

HsWnt10a DALQR--------GK----------GLSHGVPEHPALPTASPGL------QDSWEWGG**C**S 215

NvWnt10 ---------------------------------------------------TGWEWGG**C**G 158

PdWnt10 ---------------------------------------------------NQWQWKG**C**D 160

DpWnt10 -----------------------------------------NGGFGSSSTLADWRWSG**C**S 175

DmWnt10 KKQFL-----------------------QYLETNQILTPEEEKKYERSKIASRWKWGG**C**S 180

BnWnt10 --------------------------------------------------GNEFGWNG**C**X 165

HsWnt6b HDMDFGEKFSRDFLDSREAPRDIQARMRIHNNRVGRQVVTENLKRK**C**K**C**HGTSGS**C**QFKT 260

DrWnt10b HDIRFGVRFSRDWLDSRGSPRDIHARTRIHNNRVGRQVVTDNMRRK**C**K**C**HGTSGS**C**QFKT 286

HsWnt6a PDMGFGERFSKDFLDSREPHRDIHARMRLHNNRVGRQAVMENMRRK**C**K**C**HGTSGS**C**QLKT 275

NvWnt10 DNIDYGIETSAKFLDSREKGRDLHSMMNMHNNMVGRTTLSENAKTK**C**K**C**HGMCGS**C**SVKT 218

PdWnt10 HNVEFGNAFGRKFLDSEDRAKDFMSKVNRHNNKVGRMTVFENLRKM**C**KRHGMSGS**C**EMKT 220

DpWnt10 HNMDFGVKFSRFLLDSRQRGQDIHSRIHLHNSHVGRTVVGANSEIR**C**K**C**HGMSGS**C**EMKT 235

DmWnt10 HNMDFGVEYSKLFLDCREKAGDIQSKINLHNNHAGRIAVSNNMEFR**C**K**C**HGMSGS**C**QLKT 240

BnWnt10 HDLEFGAKYSRKFLQQKNKDXSIHSLMKAHNSKIGRKAXXDQKSTK**C**K**C**HGMSSS**C**EIKT 225

HsWnt6b **C**WRAAPEFRAVGAALRERLGRAIFIDTHNRNS-------------------GAFQPR--- 298

DrWnt10b **C**WYVSPEFRLVGSLLREKFLTAIFINSQNKNN-------------------GVFNSRTGG 327

HsWnt6a **C**WQVTPEFRTVGALLRSRFHRATLIRPHNRNG-------------------GQLEPGPAG 316

NvWnt10 **C**WKTVPDIREIGDRLMEKYDHATTIGMGNGRL-------------------RLHLTR--- 256

PdWnt10 **C**WRAAPQFHVVGEVLKQKYLQASKVQMINTNS-------------------ASGRVRLRL 261

DpWnt10 **C**WKAVPEFRRVGTVLKERFNQAVLVDQSQLGN-------------------EAGSSS--- 273

DmWnt10 **C**WKSAPDFHIVGKVLKHQFRKAILVDQSNLGNGEPVVVLKRARNKKSNGGSGSGSTSPDL 300

BnWnt10 **C**WLAAPDLKEVGDKLRNLYENSIQVDRTNSIQ-------------------GKITPQLAI 266

HsWnt6b -----------------------------------------LRPRRLSGELVYFEKSPDF 317

DrWnt10b STGSDP--------------------------------LRGQRRRSISRELVYFEKSPDF 355

HsWnt6a APSPAPG-------------------------------APGPRRRASPADLVYFEKSPDF 345

NvWnt10 ---------------------------------------RKARRSSVGRALVYYEDSPNY 277

PdWnt10 VYK------------------------------------KKRRKRPSKSSLVFYETSPNF 285

DpWnt10 -----------------------------------KGRQFARRIR--DTDLLFYERSPNF 296

DmWnt10 DSTDASGGHDDGGTGDSETRRHDELGVERGTRQPSADKNAARMARKLETSLFYYQRSPNF 360

BnWnt10 EDNSVS---------------------------------KRPSQKSLKKSMVYYENSPTF 293

HsWnt6b **C**ERDPTMGSPGTRGRA**C**NKTSRLLDG-**C**GSL**CC**GRGHNVLRQTRVER**C**H**C**RFHW**CC**YVL**C** 376

DrWnt10b **C**DREPAVDSLGTQGRI**C**NKSSPGMDG-**C**GSL**CC**GRGHNILKQARSER**C**H**C**RFHW**CC**YVL**C** 414

HsWnt6a **C**EREPRLDSAGTVGRL**C**NKSSAGSDG-**C**GSM**CC**GRGHNILRQTRSER**C**H**C**RFHW**CC**FVV**C** 404

NvWnt10 **C**IENKELGIFGTRGRI**C**SPESLDTDN-**C**QNL**CC**ERGYTTKKLQVTKR**C**R**C**HFSWW**C**YLI**C** 336

PdWnt10 **C**EDSSWLDSPGTRGRY**C**NKTSTDIDN-**C**ETL**CC**GRGYNTLKVTRVER**C**N**C**RFHW**CC**YVV**C** 344

DpWnt10 **C**EERPDVDYPGITGRR**C**NKTGDELDN-**C**QSL**CC**GRGYNVVRQKRTER**C**H**C**RFHW**CC**SVV**C** 355

DmWnt10 **C**ERDLGADIQGTVGRK**C**NRNTTTSDG-**C**TSL**CC**GRGHSQVIQRRAER**C**H**C**KFQW**CC**NVE**C** 419

BnWnt10 **C**DTIQEIGSYGTRGRV**C**NNTSTAAENT**C**SAL**CC**GRGFFVVRVHRVEK**C**N**C**RFHW**CC**YVE**C** 353

HsWnt6b DE**C**KVTEWVNV**C**K 389

DrWnt10b EE**C**KVTEWVNV**C**K 427

HsWnt6a EE**C**RITEWVSV**C**K 417

NvWnt10 DT**C**RETSTVSI**C**S 349

PdWnt10 KK**C**LISDWVTV**C**K 357

DpWnt10 NN**C**TVEQWVTV**C**K 368

DmWnt10 EE**C**HVEEWISI**C**N 432

BnWnt10 QK**C**EYDEWVTV**C**K 366
